# The prognostic biomarker L-homoarginine is a substrate of the cationic amino acid transporters CAT1, CAT2A and CAT2B

**DOI:** 10.1038/s41598-017-04965-2

**Published:** 2017-07-06

**Authors:** Anja Chafai, Martin F. Fromm, Jörg König, Renke Maas

**Affiliations:** Chair of Clinical Pharmacology and Clinical Toxicology, Institute of Experimental and Clinical Pharmacology and Toxicology, Friedrich-Alexander-Universität Erlangen-Nürnberg, Erlangen, Germany

## Abstract

Low plasma concentration of L-homoarginine is an independent predictor of cardiovascular events and total mortality. Experimental data indicate that supplementation of L-homoarginine may have protective effects. We aimed to elucidate the mechanisms involved in the cellular uptake of L-homoarginine, which are little understood, so far. Using human embryonic kidney (HEK293) cell lines stably overexpressing the human cationic amino acid transporters CAT1 [solute carrier family 7 (*SLC7A1*)], CAT2A (*SLC7A2A*) or CAT2B (*SLC7A2B*) we assessed the transport kinetics of L-homoarginine and interactions with the CAT substrates L-arginine and asymmetric dimethylarginine (ADMA). Significant uptake of L-homoarginine was observed for all three CATs with apparent K_M_-values of 175 ± 7 µM for CAT1 and 523 ± 35 µM for CAT2B. Saturation of CAT2A-mediated L-homoarginine uptake could not be reached. Uptake of L-homoarginine by any of the three CATs could be inhibited by L-arginine and ADMA. Significant inhibition of CAT1-mediated uptake of L-homoarginine by L-arginine already occurred in the physiological concentration range. Taken together these data demonstrate that L-homoarginine is a substrate of CAT1, CAT2A and CAT2B and that CAT1 is a key site with regard to physiological relevance and interactions with related substrates such as L-arginine.

## Introduction

Prospective clinical studies across different populations have characterized L-homoarginine as an independent protective biomarker^1^. The higher its plasma concentration the lower the cardiovascular event rate and total mortality^2–5^. This inverse association with risk sets L-homoarginine apart from numerous other risk markers, which usually show an increase in risk with increasing plasma concentrations. A high urinary excretion of L-homoarginine is associated with lower all-cause mortality in renal transplant recipients^6^. Moreover, plasma L-homoarginine tends to be lower in renal failure^2, 4^ much in contrast to structurally related biomarkers such as asymmetric dimethylarginine (ADMA), which typically increases in renal failure^7^. In animal experiments supplementation of L-homoarginine showed protective effects such as improved neurological outcome after experimental stroke, indicating that L-homoarginine may be a protective factor rather than a protective marker^8–10^. Among others, L-homoarginine is a substrate of nitric oxide synthases^11, 12^ and arginases^13^. In both cases, it has a lower affinity to these enzymes than L-arginine, which may turn it into an effective inhibitor in the presence of L-arginine. Therefore, interactions with the L-arginine-NO pathway have been considered as a possible mechanism to explain its clinical effects^2, 8^. But L-homoarginine may also affect L-arginine independent functions as it is known to inhibit the alkaline phosphatase(s)^14^ and the Na^+^/K^+^-ATPase^15^. Irrespective of prevailing uncertainties regarding the precise mechanisms mediating its putative beneficial effects, supplementation of L-homoarginine has already become the target of first clinical investigations^16^.

L-Homoarginine is formed endogenously and may also be ingested as part of the diet^17^. While major pathways and enzymes involved in its synthesis, metabolism and excretion have been identified^8, 11, 18^ the precise mechanisms underlying its distribution in the body and its uptake by cells remain only partly understood. Cationic amino acids such as L-homoarginine largely depend on transport proteins to cross cellular membranes^19^. In early experiments it has been recognized that L-homoarginine competes with the cellular uptake of L-arginine mediated by the so called y^+^-system^20–22^. This makes the cationic amino acid transporters of the CAT family (i.e. solute carrier 7 family – *SLC7*), which account for the transport activity of the y^+^-system, primary targets for further investigations. For CAT1 (*SLC7A1*), CAT2A (*SLC7A2A*) and CAT2B (*SLC7A2B*) direct evidence for transport of L-arginine and methylarginines is available^19, 23, 24^, which makes them promising candidates for the investigation of L-homoarginine transport. The CATs differ in their tissue distribution^19^. CAT1 is widely expressed in the body, with the exception of the liver. In contrast, CAT2A is predominantly expressed in the liver, while CAT2B expression appears to be functionally relevant in immune cells. In the brain immunostaining for CAT1, CAT2 and CAT3 has been observed^25^.

So far, data regarding putative transport of L-homoarginine by the y^+^-system/CATs are mostly based on assays in native cells representing the net effect of multiple related transport systems and/or on indirect evidence such as using L-homoarginine as an inhibitor or unlabeled substrate for trans-stimulation (i.e. exchange)^20, 22, 26^. In order to correctly interpret physiological effects of L-homoarginine and to gauge possible side effects of L-homoarginine supplementation it is of considerable interest to identify transport proteins involved in the cellular uptake of L-homoarginine and to know whether L-homoarginine is likely to interact with the transport of related substrates such as L-arginine or ADMA. Therefore, it was the aim of the present study to assess transport mechanisms involved in the cellular uptake of L-homoarginine focusing on the cationic amino acid transporters CAT1, CAT2A and CAT2B. Furthermore, possible interactions of CAT-mediated L-homoarginine uptake with known CAT substrates were investigated. Cellular uptake was studied in human embryonic kidney derived cell lines (HEK293) stably overexpressing CAT1, CAT2A or CAT2B. In previous studies we found the HEK cell lines well suited to study the CAT1-, CAT2A- or CAT2B-mediated cellular uptake of substrates related to homoarginine (such as L-arginine and ADMA)^23, 24^. In addition, using the same cell lines for L-homoarginine makes it easier to compare the present data for L-homoarginine to data published for L-arginine and ADMA.

## Results

### CAT1-, CAT2A- and CAT2B-mediated uptake of L-homoarginine

**Figure 1:**
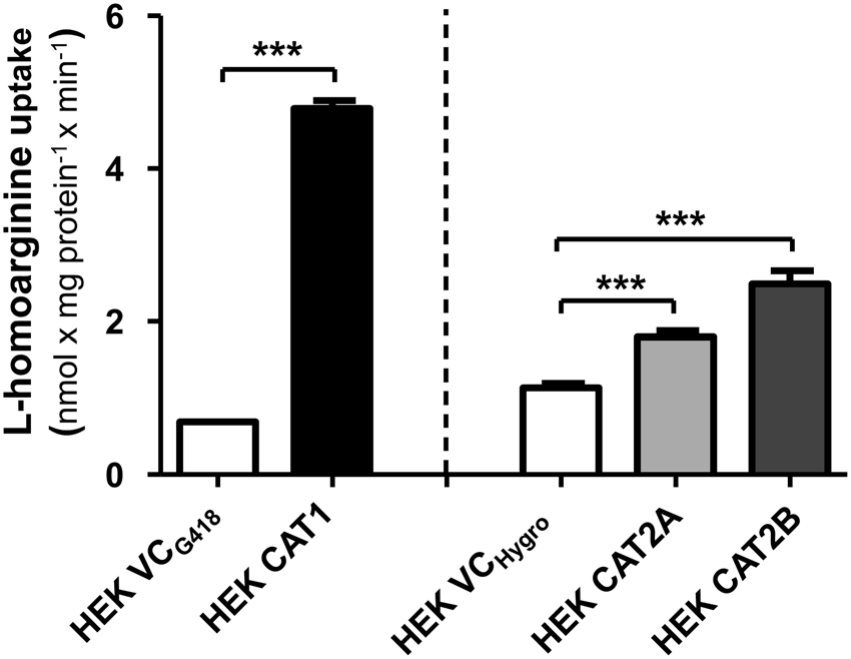
Uptake of L-homoarginine (100 µmol/l) by HEK cells overexpressing CAT1 (HEK CAT1), CAT2A (HEK CAT2A), CAT2B (HEK CAT2B) and by the respective control cells (HEK VC_G418_ or HEK VC_Hygro_). Each data point is n = 6, mean ± SEM. ***p < 0.001.

**Figure 2:**
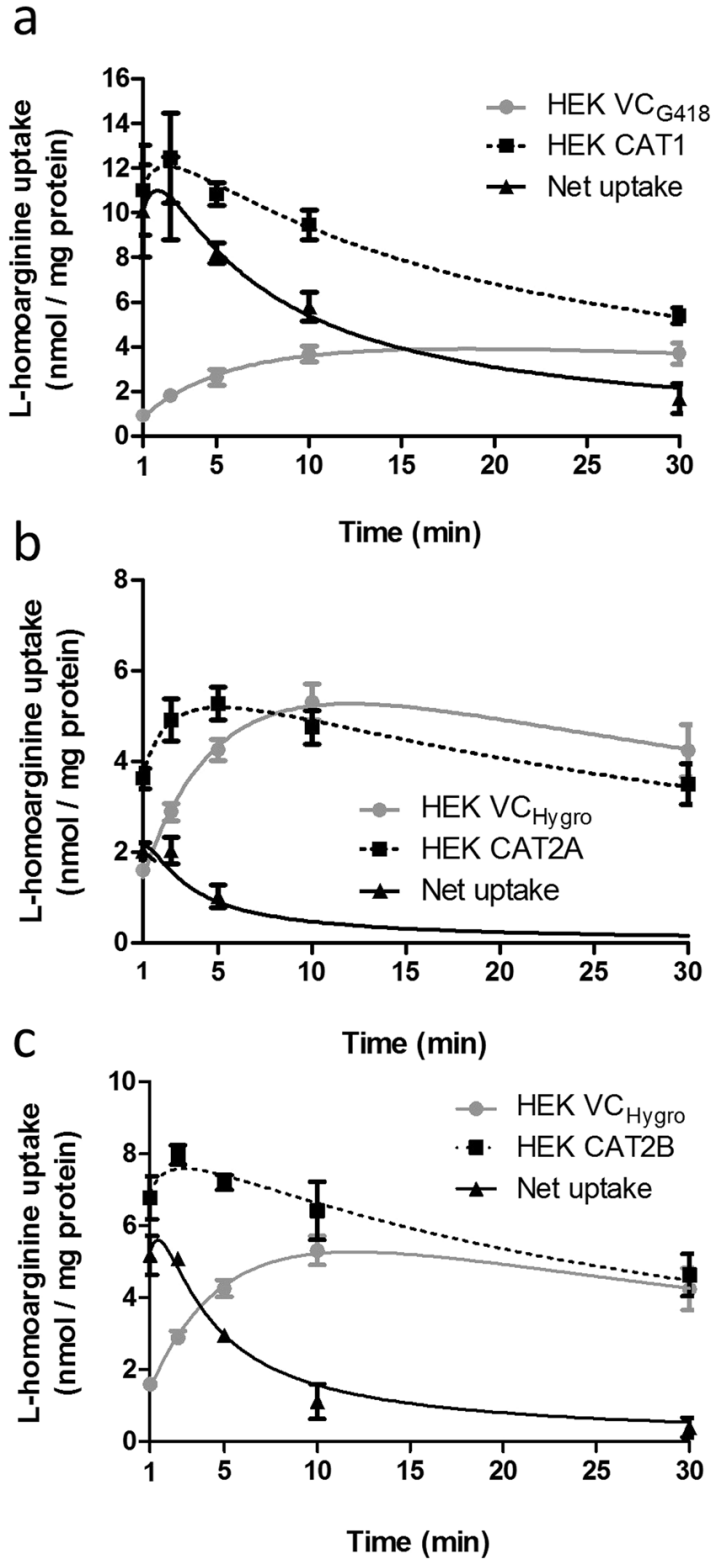
Time-dependent uptake of L-homoarginine in HEK cells overexpressing CAT1 (**a**), CAT2A (**b**) or CAT2B (**c**). Cells were incubated for 1, 2.5, 5, 10, and 30 min at 37 °C with 100 μM L-homoarginine. Each data point is n = 6. ***p < 0.001 as compared to suitable cells (HEK VC_G418_ or HEK VC_Hygro_) containing only an empty vector.

**Figure 3:**
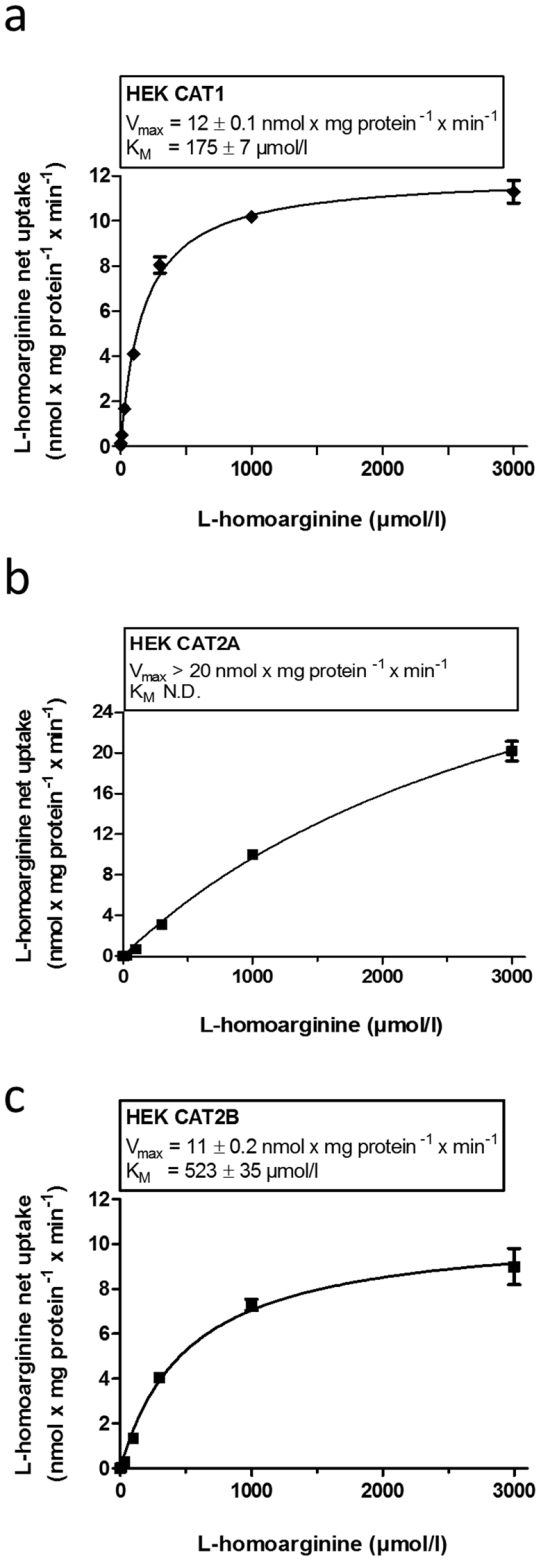
CAT1- (**a**), CAT2A - (**b**) or CAT2B-mediated (**c**) uptake of L-homoarginine dependent of the L-homoarginine concentration. Each data point is n = 6.

In experiments using L-homoarginine (100 µmol/l) as substrate, cellular uptake was significantly higher in HEK cells overexpressing CAT1 (7-fold), CAT2A (1.6-fold) or CAT2B (2.2-fold), respectively, as compared to the uptake into control cells (p < 0.001 vs. HEK VC_G418_ or HEK_Hygro_, Fig. 1). Next, time dependence of L-homoarginine uptake was assessed. As shown in Fig. 2 uptake of L-homoarginine was higher in CAT1-, CAT2A- or CAT2B-expressing cells than in the respective control cells over periods of 30 min, 5 min, and 30 min, respectively. For all cell lines linearity of uptake was observed up to 2.5 min, which was chosen as incubation period for subsequent experiments. The CAT1-, CAT2A- or CAT2B-mediated net uptake was used to calculate K_M_- and V_max_- values (Fig. 3). The apparent V_max_-values for CAT1 and CAT2B-mediated uptake of L-homoarginine were 12 ± 0.1, and 11 ± 0.2 nmol × mg protein^−1^ × min^−1^, respectively. The corresponding K_M_-values for CAT1 and CAT2B, were 175 ± 7 µmol/l and 523 ± 35 µmol/l, respectively. The V_max_-value for CAT2A could only be estimated to be >20 nmol × mg protein^−1^ × min^−1^ due to the technical limit imposed by the maximally attainable L-homoarginine concentration.

### Inhibition of CAT1-, CAT2A- and CAT2B-mediated L-homoarginine uptake by L-arginine and ADMA

**Figure 4:**
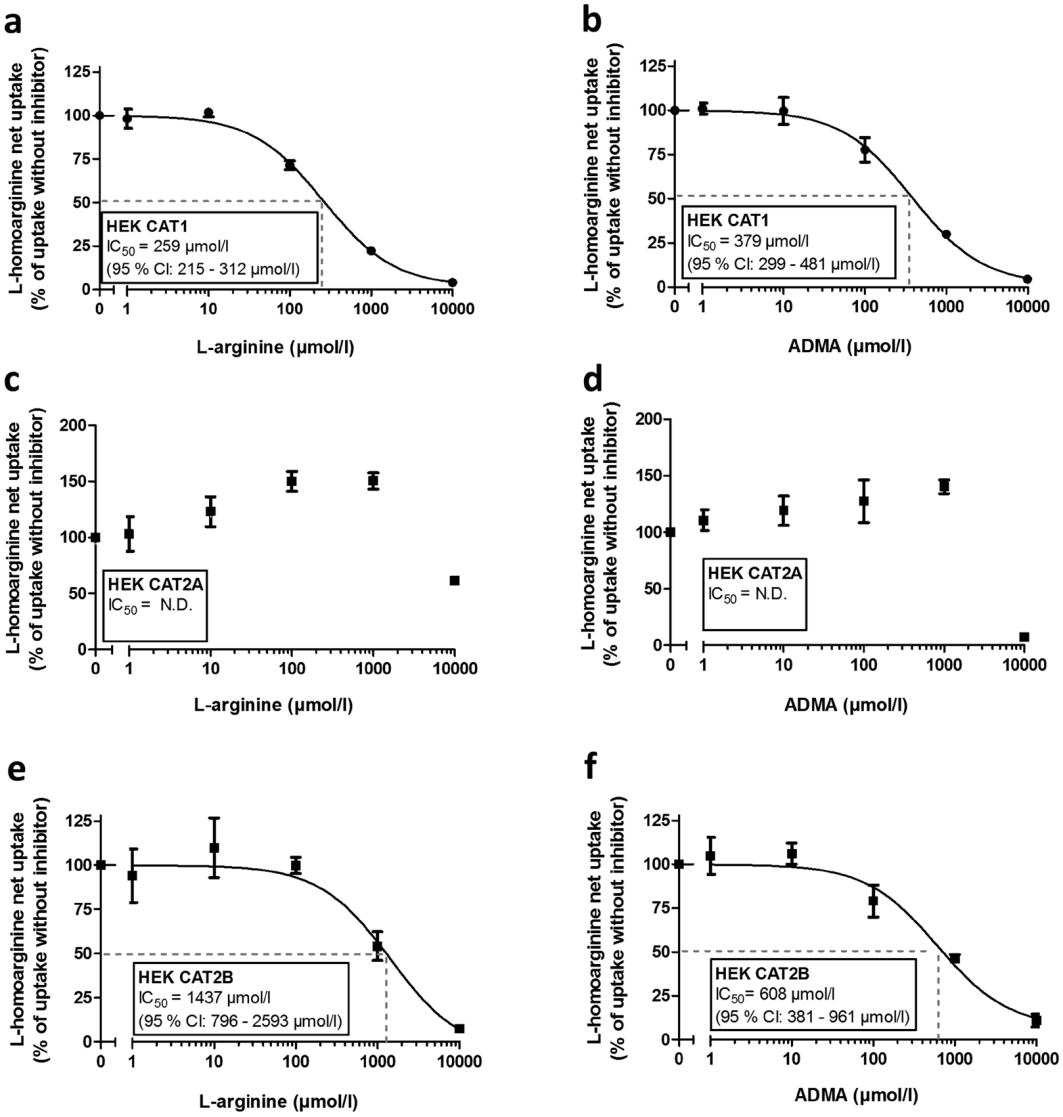
Inhibition of CAT1-, CAT2A-, CAT2B-mediated L-homoarginine (100 µmol/l) uptake by increasing concentrations of L-arginine (**a**,**c**,**e**) and ADMA (**b**,**d**,**f**). Each data point is n = 6.

We investigated the inhibition of CAT1-, CAT2A- and CAT2B-mediated uptake of L-homoarginine (100 µmol/l) by L-arginine and ADMA, which both are established and structurally related substrates of these transporters (Fig. 4). CAT1-mediated uptake of L-homoarginine was inhibited by increasing concentrations of L-arginine and ADMA with IC_50_-values of 259 µmol/l (95% CI: 215–312 μmol/l) and 379 μmol/l (95% CI: 299–481 μmol/l), respectively (Fig. 4a,b). CAT2A-mediated transport of L-homoarginine could only be inhibited by very high (10000 µmol/l) concentrations of L-arginine and ADMA which precluded the calculation of IC_50_-values (Fig. 4c,d). For inhibition of CAT2B-mediated uptake of L-homoarginine the IC_50_-values were 1437 µmol/l (95% CI: 796–2593 μmol/l) and 608 μmol/l (95% CI: 381–961 μmol/l) for L-arginine and ADMA, respectively (Fig. 4e,f).

**Figure 5:**
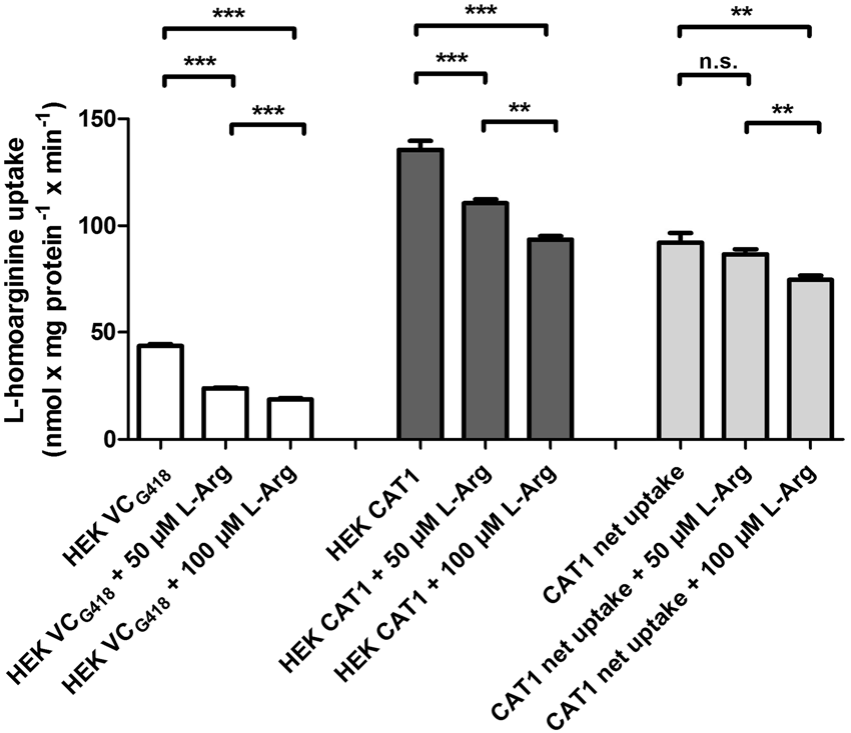
Inhibition of L-homoarginine uptake by HEK CAT1 cells and respective control cells (HEK VC_G418_) and resulting CAT1-mediated net uptake without and with co-administration of L-arginine in physiological concentrations. Each data point is n = 6, mean ± SEM, ***p < 0.001, **p < 0.01.

As the IC_50_-value of CAT1-mediated L-homoarginine uptake by L-arginine was close to the physiological concentration range of L-arginine (ranging from a mean of 77 µmol/l in fasting individuals^27^ to up to 115 µmol/l post prandial^28^) we assessed the effect of L-arginine (50 and 100 µmol/l) on CAT1-mediated uptake of L-homoarginine (2 µmol/l) in the physiological concentration range of both substances (Fig. 5). CAT1-mediated net uptake of L-homoarginine was significantly inhibited by 100 µmol/l L-arginine (p < 0.01).

### L-Homoarginine as inhibitor of CAT1-, CAT2A- and CAT2B-mediated L-arginine and ADMA uptake

**Figure 6:**
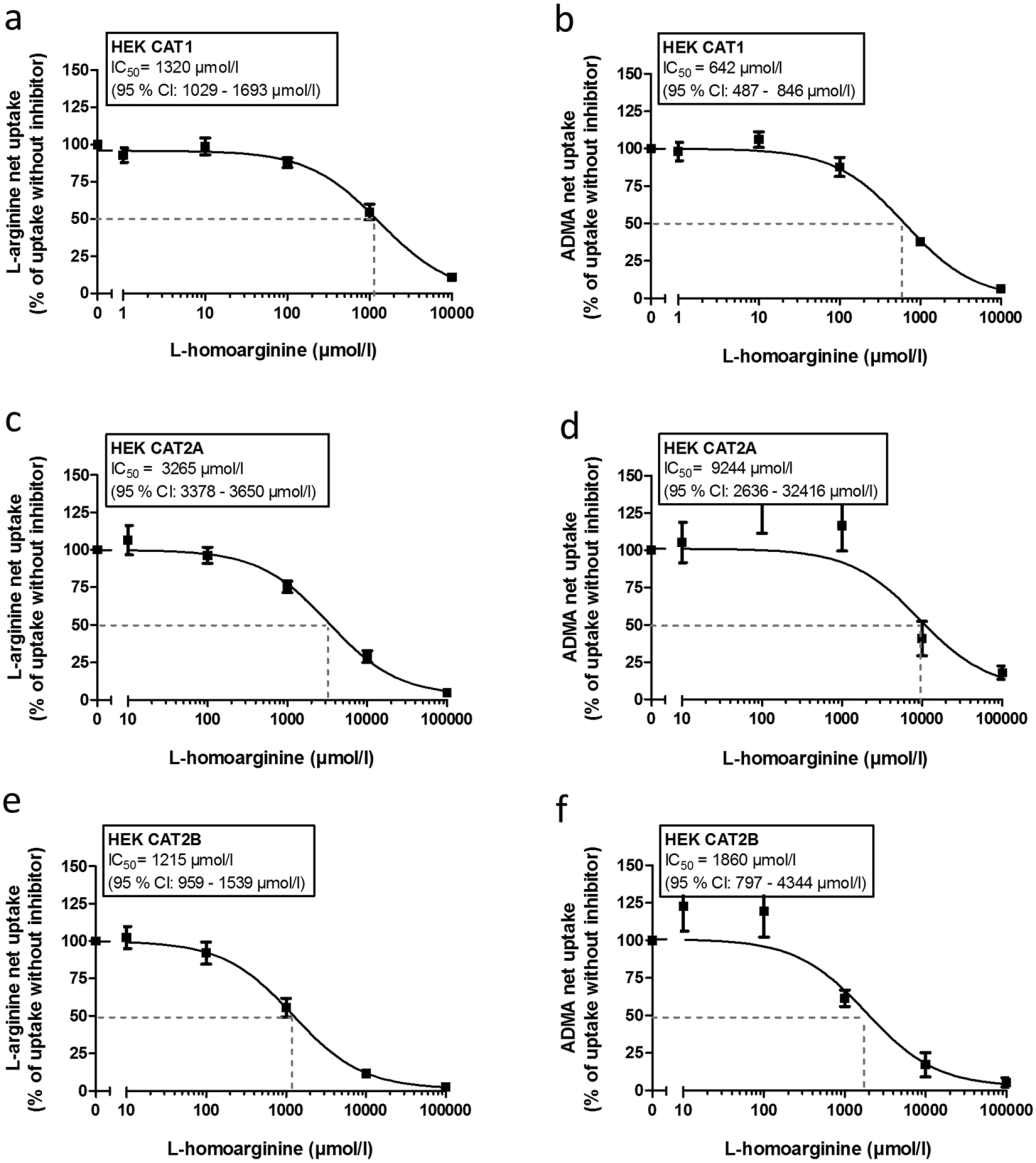
Inhibition of CAT1-, CAT2A-, CAT2B-mediated L-arginine (**a**,**c**,**e**) and ADMA (**b**,**d**,**f**) uptake by increasing concentrations of L-homoarginine. Each data point is n = 6.

As substrate of CAT1, CAT2A and CAT2B L-homoarginine may also inhibit the uptake of other CAT substrates. We therefore assessed the inhibition of CAT1-, CAT2A- and CAT2B-mediated L-arginine or ADMA uptake (Fig. 6). CAT1-mediated uptake of L-arginine and ADMA was inhibited by increasing L-homoarginine concentrations with IC_50_-values of 1320 μmol/l (95% CI: 1029–1693 μmol/l) and 642 μmol/l (95% CI: 487–846 μmol/l), respectively (Fig. 6a,b). IC_50_-values for inhibition of CAT2A-mediated uptake of L-arginine and ADMA were 3265 μmol/l (95% CI: 3378–3650 μmol/l) and 9244 μmol/l (95% CI: 2636–32416 μmol/l), respectively (Fig. 6c,d), while IC_50_-values for inhibition of CAT2B-mediated uptake of L-arginine and ADMA by L-homoarginine were lower with 1215 μmol/l (95% CI: 959–1539 μmol/l) and 1860 μmol/l (95% CI: 797–4344 μmol/l), respectively (Fig. 6e,f).

## Discussion

It was the aim of the present study to characterize the cellular uptake of the protective biomarker L-homoarginine by CATs and to assess the potential relevance of interactions with related endogenous CAT substrates. Transport kinetics were assessed using HEK293 cells stably overexpressing human CAT1, CAT2A and CAT2B. Our principal new findings are:

1. L-Homoarginine is a substrate of the human cationic amino acid transporters CAT1, CAT2A and CAT2B.
2. CAT1-, CAT2A- and CAT2B-mediated uptake of L-homoarginine can be inhibited by L-arginine and ADMA, but only inhibition of CAT1-mediated uptake of L-homoarginine is likely to be functionally relevant under physiological conditions.
3. CAT1-, CAT2A- and CAT2B-mediated uptake of L-arginine and ADMA can be inhibited by L-homoarginine, but this is unlikely to be clinically relevant in the physiological concentration range.

### CAT1-, CAT2A- and CAT2B-mediated uptake of L-homoarginine

Considering the observed low K_M_-values and the known broad tissue distribution^19^ CAT1 likely is the most relevant of the three CATs with regard to translocation through cell membranes and the biological effects of L-homoarginine. Relevant CAT1-mediated uptake of L-homoarginine was already observed at concentrations (2 µmol/l) in the (patho-) physiological concentration range of L-homoarginine (ranging from 1.5 to 2.4 µmol/l in healthy volunteers^29^ to up to 4.7 µmol/l in women with preeclampsia^30^), which is still below the apparent K_M_-value of 175 µmol/l of CAT1-mediated L-homoarginine transport.

With respect to possible side effects of clinical arginine supplementation it is of interest to note that in previous studies the L-homoarginine concentration required to attenuate L-arginine-dependent NO synthesis^22, 31^ was several orders of magnitude above the IC_50_-values we observed for inhibition of CAT1- or CAT2B-mediated uptake of L-arginine by L-homoarginine. Moreover, the IC_50_-values are far above the physiological plasma concentration of L-homoarginine^5, 16^. All this makes a significant interference of oral L-homoarginine supplementation with cellular uptake of L-arginine and NOS synthesis unlikely. In animals orally supplemented L-homoarginine is readily absorbed in the small intestine and largely excreted unmetabolized^32^. Oral supplementation of L-homoarginine in humans leads to a significant and quite persistent rise in plasma L-homoarginine^16^ indicating rather limited metabolism. This low rate of systemic metabolism still awaits explanation as several enzymes including NOS, arginase and alanine-glyoxylate aminotransferase 2 (AGXT2) are capable of metabolizing L-homoarginine^11–13, 18^. The simplest explanation is that between absorption and renal elimination only a small fraction of L-homoarginine is taken up by metabolizing tissues such as the liver. Our data which indicate a low affinity, high capacity transport of L-homoarginine by CAT2A are in line with this. We found CAT2A-mediated uptake of L-homoarginine to be detectable even at physiological L-homoarginine concentrations that are several orders of magnitude below its presumed K_M_-values. Uptake at these concentrations was quantitatively limited, however. Similar observations have been made for CAT2A-mediated transport of L-arginine^24^. Moreover, the pharmacokinetics of L-homoarginine in supplementation studies, with a large proportion of the ingested L-homoarginine being excreted unmetabolized, indicate that overall uptake of L-homoarginine into the liver may be limited and/or saturable. The expression pattern of CAT2 and functional considerations may explain this observation: In humans the liver is a key site of CAT2A expression, it is also an organ with a high enzymatic capacity for L-arginine and L-homoarginine metabolism^33^. High affinity uptake at low concentration by the liver and subsequent metabolism would lead to a functionally relevant depletion of plasma from L-arginine or L-homoarginine which hardly could be compensated by dietary intake.

### Interactions of CAT-mediated uptake of L-homoarginine, L-arginine and ADMA

While L-homoarginine has already been used for over 35 years as a competitive inhibitor of the y^+^-mediated transport of cationic amino acids^20^, its interaction with individual components of this system (i.e. CAT1, CAT2A and CAT2B) still remained to be elucidated. The present study indicates that the high concentrations of L-homoarginine (1–10 mM) typically used in these studies^20–22, 31^ can indeed inhibit CAT-mediated transport of substrates, such as L-arginine. In a previous study it was noted that despite being a NO substrate, L-homoarginine in high doses actually inhibited NO synthesis in perfused rat kidneys^31^. Based on the observed effects of siRNA-mediated knockdown of CAT1 expression the observation was attributed to inhibition of CAT1-mediated L-arginine uptake resulting in cellular depletion of the superior NOS substrate L-arginine. This interpretation is supported by the present data. In light of the controversial discussion regarding the risks and benefits associated with L-arginine supplementation^34, 35^, it will be of interest to further investigate the clinical relevance of this interaction by studying samples available from L-arginine supplementation studies. In this repect possible interactions at the intracellular (i.e. mitochondrial) transport level involving *SLC25A29* (encoding ORNT3) will have to be considered as well^36^.

With respect to biological effects of L-homoarginine it was also of interest to investigate a possible interference of L-homoarginine with the CAT1-, CAT2A- or CAT2B-mediated uptake of related substances such as L-arginine and ADMA. The present *in vitro* findings indicate that *in vivo* CAT1-, CAT2A- or CAT2B-mediated transport of L-arginine or ADMA is unlikely to be affected in a clinically significant manner by physiological concentrations of L-homoarginine. This observation is consistent with very recent observations in a clinical study in which no effect of L-homoarginine on the plasma concentrations of L-arginine and ADMA and on vascular function was observed when mean plasma concentrations of healthy volunteers were augmented from 2.9 to 17.3 µmol/l^16^.

However, in line with previous observations concerning the CAT substrate ADMA^23, 24^ our data show that physiological concentrations of L-arginine may affect the CAT1-, CAT2A- or CAT2B-mediated transport of L-homoarginine. Based on this observation one would expect, in principle, that supplementation of L-arginine leads to increased plasma L-homoarginine concentrations and lowers intracellular L-homoarginine. But similar to the previously reported effects of L-arginine on the cellular uptake of ADMA, the consequences of L-arginine supplementation on plasma L-homoarginine can be expected to be modest. The IC_50_-value for inhibition of CAT1-mediated L-homoarginine uptake by L-arginine is 259 µM, which is only approached by specific dietary supplementations^37, 38^. However, we already previously noted that *in vivo* CAT1 is simultaneously confronted with several chemically similar substrates competing for transport (including ADMA, monomethylarginine, L-arginine, L-lysine, L-histidine - and now L-homoarginine) which may give each substrate more apparent leverage at lower concentrations^23^.

The primary focus of the present study was CAT-mediated cellular uptake of L-homoarginine and the experiments were designed accordingly. Based on the data from the literature for related substrates and on our own preliminary data indicating a high transport capacity, concentrations above the physiological concentration range were required to determine some of the K_M_-, V_max_- and IC_50_- values for homoarginine and ADMA. In addition, assays performed at higher concentrations are less sensitive to metabolism and interference by endogenous compounds. Furthermore, considering the short incubation time used in the present experiments, the extent and impact of metabolism on the observed transport kinetics can be considered marginal.

In the literature K_M_-values for the uptake of L-homoarginine by cells or tissues, rather than for individual transport proteins, can be found. These range from 40 µmol/l in fibroblasts^20^ up to 300 µmol/l in rat hepatocytes^39^. The IC_50_-values reported in the literature for inhibition of y^+^-system-mediated uptake of L-arginine by L-homoarginine range between 19 µmol/l and 240 µmol/l, respectively^20, 21^. This is much lower than the IC_50_-value of 1320 µmol/l we observed for the inhibition of CAT1-mediated uptake of L-arginine by L-homoarginine. The differences between data obtained for cell types and the values we obtained for individual transport proteins highlight the need to dissect the individual components of transport systems and to look beyond CATs for y^+^-like transport of L-homoarginine and arginine.

Alternative candidates for transport of L-homoarginine are plentiful. Transporters capable of transporting L-arginine or ADMA may serve as a good starting point in this respect. Experimental and clinical data show that the y^+^LAT system is involved in the cellular extrusion of L-arginine and ADMA^40^. A similar role of the y^+^LAT system in extrusion of L-homoarginine can be presumed but remains to be formally proven. In addition, the multidrug and toxin extrusion protein 1 (MATE1, *SLC47A1*) and the organic cation transporter 2 (OCT2, *SLC22A2*) can be considered as candidates for the cellular uptake of L-homoarginine^24^.

In principle, genomewide association studies (GWAS) may help to identify transport proteins relevant in the homeostasis of plasma L-homoarginine. However, no decisive evidence indicating the association of plasma L-homoarginine with specific transport proteins has emerged in these studies, so far^8, 41^. GWAS data available for L-arginine and ADMA point, with respect to transport, into the same direction^42^. These findings are compatible with several constellations which are not mutually exclusive:

1. Redundant transport systems render the contribution of an individual transport protein too small to emerge as significant in GWAS analyses.
2. Transport functionality for L-homoarginine or a related co-substrate is vital, which keeps the frequency of functionally relevant genetic variations below the limit of detection in GWAS analyses.
3. For key transport proteins involved in the transport of L-homoarginine there are no frequent and functionally relevant SNPs to be found in the general population.

## Conclusion

The present study identifies L-homoarginine as substrate of CAT1, CAT2A and CAT2B. Especially uptake by CAT1 and interactions with the uptake of L-arginine are likely to be of (patho-) physiological relevance.

## Material and Methods

### Chemicals

[^3^H]-L-Homoarginine (6 Ci/mmol, >99% radiochemical purity, generally labeled, ^3^H[G]) was obtained from ViTrax Co (CA, USA), [^3^H]-ADMA (25 Ci/mmol, >97% radiochemical purity, ^3^H[G] labeled by catalytic tritium gas exchange) was from BIOTREND Chemikalien GmbH (Cologne, Germany) and [2,3,4,5-^3^H]‐labeled L-arginine (43 Ci/mmol, >99% radiochemical purity) was from American Radiolabeled Chemicals Inc. (St. Louis, MO, USA). Stock solutions of [^3^H]-labeled L-homoarginine, ADMA and L-arginine in water contained 20%, 50% and 2% ethanol, respectively. Unlabeled L‐arginine and ADMA were from Enzo Life Sciences GmbH (Lörrach, Germany). Sodiumbutyrate was obtained from Merck KGaA (Darmstadt, Germany). Poly-D-lysine hydrobromide (research grade, molecular weight 70,000–150,000) and unlabeled L-homoarginine were purchased from Sigma-Aldrich Chemie GmbH (Taufkirchen, Germany). Unless stated otherwise purity of unlabeled compounds was as least ≥97%.

### Cell culture

Cell lines and basic cell culture conditions have previously been described^23, 24^. The original choice of human embryonic kidney cells (HEK293) cells for transfection with CAT1-, CAT2A- or CAT2B- was based on their reliable growth and well established propensity for transfection and cellular uptake experiments. In brief, uptake assays were performed using [^3^H]-labeled substrate and human embryonic kidney (HEK293) cells stably overexpressing the empty vector only (HEK VC_G418_ and HEK VC_hygro_, depending on selection conditions) or human CAT1 (HEK CAT1, gene: *SLC7A1*, NM_003045.4), CAT2A (HEK CAT2A, gene: *SLC7A2A*, NM_003046.5) or CAT2B (HEK CAT2B, *SLC7A2B*, NM_001008539.3). The cells were cultured in minimal essential medium containing 10% heat-inactivated fetal bovine serum, 100 U/l penicillin, 100 μg/ml streptomycin, and either G418 (800 µg/ml) or hygromycin B (260 µg/ml) at 37 °C and 5% CO_2_ with subculture as required. Trypsin (0.05%)-EDTA (0.02%) solution was used to detach cells. All cell culture media supplements were obtained from Invitrogen GmbH (Karlsruhe, Germany).

### Uptake transport assays

The transport assays were performed as previously described with minor modifications^23, 24^. Cells were seeded into poly-D-lysine-coated 12-well plates. Sodium butyrate (10 mmol/l) was added after 24 h of culture for 24 h to enhance CAT1, CAT2A or CAT2B protein expression. For the uptake experiments cells were washed with prewarmed (37 °C) transport buffer (142 mmol/l NaCl, 5 mmol/l KCl, 1 mmol/l K_2_HPO_4_, 1.2 mmol/l MgSO_4_, 1.5 mmol/l CaCl_2_, 5 mmol/l glucose, and 12.5 mmol/l HEPES, pH 7.3) and incubated with a mixture of radiolabeled and non-radiolabeled L-homoarginine, L-arginine or ADMA in transport buffer at 37 °C. In order to identify the period of linear L-homoarginine uptake time dependence was assessed. Cells were incubated with the transport buffer containing 100 μmol/L L-homoarginine at 37 °C for 1, 2.5, 5, 10 and 30 min. For determination of kinetic parameters of CAT-mediated L-homoarginine transport increasing L-homoarginine concentrations and an incubation time of 2.5 min were used.

To analyze inhibition of CAT-mediated uptake of L-homoarginine by L-arginine or ADMA as well as CAT-mediated uptake of L-arginine and ADMA by L-homoarginine, cells were co-incubated with different concentrations of the potential inhibitors. For the uptake of L-homoarginine (100 µmol/l) inhibitor concentrations from 1-10000 µmol/l of L-arginine and ADMA were used. For the inhibition of the uptake of ADMA (100 µmol/l) or L-arginine (1000 µmol/l) L-homoarginine concentrations from 1–100000 µmol/l were used. To analyze inhibition of CAT1-mediated uptake of L-homoarginine at physiological concentrations, a substrate concentration of 2 µmol/l and an inhibitor concentration of up to 100 µmol/l for L-arginine was used. After cooling to 0 °C the cells were washed three times with ice-cold transport buffer and lysed with 0.2% SDS. The intracellular accumulation of radioactivity was determined by liquid scintillation counting (TriCarb 2800; PerkinElmer Life and Analytical Sciences, Inc.) after adding 4 ml of scintillation solution (Ultima Gold XR; PerkinElmer Life and Analytical Sciences, Inc., Rodgau-Jügesheim, Germany). A bicinchoninic acid assay was used to assess the protein concentrations. Data from at least two single experiments each performed in triplicate (n ≥ 2 × 3) on different days were combined.

### Statistical analysis

Statistical analysis was performed using GraphPad Prism 5 (version 5.01, 2007, GraphPad Software, San Diego, CA, USA). Net transport data were calculated as the difference of uptake from cells transfected with the respective CAT transporter and the corresponding empty control vector. Therefore, the maximum transport velocity (V_max_) values have to be interpreted as “apparent” values. K_M_-values for the uptake transport were calculated using Michaelis–Menten enzyme kinetics. The corresponding IC_50_ values for inhibition were calculated by fitting the data to a sigmoid dose–response regression curve. Comparisons between the groups were done using two-tailed unpaired student t-tests and between more than two groups with one-way ANOVA. Statistical significance was defined as a P value < 0.05. Unless indicated otherwise, values are reported as mean ± standard deviation.

### Data availability

The datasets generated during and/or analysed during the current study are available from the corresponding author on reasonable request.
